# Magnetic Resonance Imaging of Soft Tissue Infection with Iron Oxide Labeled Granulocytes in a Rat Model

**DOI:** 10.1371/journal.pone.0051770

**Published:** 2012-12-07

**Authors:** Hassina Baraki, Norman Zinne, Dirk Wedekind, Martin Meier, André Bleich, Silke Glage, Hans-Juergen Hedrich, Ingo Kutschka, Axel Haverich

**Affiliations:** 1 Department of Cardio-Thoracic, Transplantation and Vascular Surgery, Hannover Medical School, Hannover, Germany; 2 Institute of Laboratory Animal Science, Hannover Medical School, Hannover, Germany; University of Heidelberg Medical School, Germany

## Abstract

**Object:**

We sought to detect an acute soft tissue infection in rats by magnetic resonance imaging (MRI) using granulocytes, previously labeled with superparamagnetic particles of iron oxide (SPIO).

**Materials and Methods:**

Parasternal infection was induced by subcutaneous inoculation of Staphylococcus aureus suspension in rats. Granulocytes isolated from isogenic donor rats were labeled with SPIO. Infected rats were imaged by MRI before, 6 and 12 hours after intravenous injection of SPIO-labeled or unlabeled granulocytes. MR findings were correlated with histological analysis by Prussian blue staining and with re-isolated SPIO-labeled granulocytes from the infectious area by magnetic cell separation.

**Results:**

Susceptibility effects were present in infected sites on post-contrast T2*-weighted MR images in all animals of the experimental group. Regions of decreased signal intensity (SI) in MRI were detected at 6 hours after granulocyte administration and were more pronounced at 12 hours. SPIO-labeled granulocytes were identified by Prussian blue staining in the infected tissue and could be successfully re-isolated from the infected area by magnetic cell separation.

**Conclusion:**

The application of SPIO-labeled granulocytes in MRI offers new perspectives in diagnostic specificity and sensitifity to detect early infectious processes.

## Introduction

Infections after surgery like mediastinitis or vascular prosthetic graft infections are life-threatening complication associated with high mortality rates up to 41% [1]–[8]. A false positive diagnosis of an infection of an aortic prosthesis/mediastinitis implies a high-risk reoperation. Therefore early and reliable diagnosis is essential for the appropriate management of the infection in order to improve the prognosis of these patients. Currently, computer tomography (CT) is still the most used diagnostic tool for evaluating infectious processes in surgical departments due to its easier accessibility, but its sensitivity and specificity are unsatisfactory with 57–67% and 39–85%, respectively [4], [6]. Besides CT scans expose patients to relatively high dose of radiation and in most cases the application of iodine-containing nephrotoxic contrast agent is necessary to highlight the infectious focus [9].

Leucocyte scintigraphy is reported to provide the highest specificity for detection of infections, however it has limited spatial resolution and is hardly available in most clinics due to the application of radioactive tracers and high costs [10], [11]. Magnetic resonance imaging (MRI) allows a much better anatomical resolution than nuclear scintigraphy and provides more physiological information than computer tomography [6], [12], [13], [14]. Inflammation causes endothelial hyperpermeability allowing extravasation of leucocytes to the inflamed tissue [15], [16]. Moreover, classic contrast-enhanced MRI using gadolinium-based contrast agents indicates increased capillary permeability of the inflamed tissue causing accumulation of contrast agent in the extracellular space. However, enhancement of contrast agents can also be observed in postoperative healing processes, degenerative diseases, tumor development and traumata, rendering this technique not specific for infection [13], [17], [18].

We intended to combine the cell tracking idea used in leucocyte scintigraphy for infection with the high anatomical imaging resolution of MRI without having the drawbacks of radiation, radioactive tracers and nephrotoxicity of iodine contrast agents. Labeling of cells with iron oxide particles prior to transplantation has been described before, in order to improve detectability of cells and produce a strong contrast with surrounding tissue [19]. Iron oxide is described as a contrast agent generating changes in T2-weighted MR images due to its high relaxivity and it has been successfully implemented for imaging of cell migration and trafficking of different cell types in vivo [12], [20]–[23].

Recent studies have used iron oxide in its advanced modification as dextran-coated iron oxide magnetic nanoparticles such as superparamagnetic iron oxide (SPIO), ultrasmall superparamagnetic iron oxide (USPIO), or cross-linked iron oxide (CLIO), micron-sized iron oxide particle (MPIO) causing hypointensity in T2-weighted MR images [24]–[27]. Lee et al. detected intravenously injected and iron oxide-labeled macrophages by MRI in experimentally induced soft tissue infection in mice. Due to the low rate of circulating monocytes in peripheral blood, they obtained syngenic macrophages by a relatively invasive procedure of peritoneal washings after the intraperitoneal injection of thioglycollate [28].

The aim of the study was to identify an acute infection in MRI using SPIO-labeled granulocytes. We evaluated SPIO-labeled granulocytes in this study, since granulocytes are the first cells migrating to the infection site and due to their high proportion of circulating leukocytes [21], [22], [29].

## Materials and Methods

This study was conducted in accordance with German law for animal protection and with the European Communities Council Directive 86/609/EEC for the protection of animals used for experimental purposes. All experiments were approved by the Local Institutional Animal Care and Research Advisory committee and permitted by the local government (Lower Saxony State Office for Consumer Protection and Food, Animal Welfare Service Permit Number: O7/1255).

We used a granulocyte recipient and a granulocyte donor animal group consisting of isogenic LEW.1AR1 inbred rats in order to avoid immunological rejection reactions. The animals in the recipient group were 12 to 14 weeks old female rats (weighing 218±18 g). The granulocyte donor group consisted of 12 to 28 week old rats (weighing 248±66 g). In order to reduce the number of animals, rats of the donor group were treated with Granulocyte Colony-Stimulating Factor (G-CFS), which duplicated the number of their circulating granulocytes [30].

### Granulocyte recruitment

Peripheral Granulocytes were isolated from blood of the donor group 24 hours after G-CSF injection [31], [32]. Blood samples were purified by removal of erythrocytes by dextran sedimentation. In the next step, mononuclear cells were withdrawn by density gradient centrifugation (FicollPaqueTM Premium, GE Healthcare) [33]. Residual erythrocytes were removed (Red Blood Cell Lysis Solution, Miltenyi Biotec GmbH, Bergisch Gladbach, Germany). Thereby, granulocytes were enriched with a purity of 98% as indicated by standard flow cytometry, performed with a Fluorescein Isothiocyanate (FITC)-conjugated mouse anti-rat granulocyte monoclonal antibody (clone HIS 48; BD Pharmingen, Germany).

### Contrast Agent and Labeling

For the intracellular magnetic labeling, a two-component kit from Miltenyi Biotec GmbH (FeraTrack^TM^) was used following the manufacturer's instructions. In brief, a transfection reagent (Loading Reagent) and SPIO particles (Contrast Particles) with a mean diameter of 100 nm, consisting of an iron oxide core coated with dextran were complexed in 100 µl RPMI (Roswell Park Memorial Institute medium) for 30 min. The complexes were transferred to cell culture dishes and incubated at 37°C for 4 hours. After transfection, cells were washed twice with PBS (phosphate-buffered saline) and harvested in 0.9% NaCl solution prior to injection [34].

### Experimental Setup

All animals underwent the same experimental setup. Anesthesia was induced by 3% isoflurane inhalation in air and maintained with 1–2% isoflurane in oxygen in all rats. The chest was shaved and cleaned with 70% isopropyl alcohol. Subsequently, 0.5 ml ×10^8^ CFU/ml of Staphylococcus aureus (*S. aureus*) suspension was inoculated subcutaneously into the left parasternal chest area of all rats by using a 25 gauge ×40 mm needle. Each rat underwent three MRI sessions. The first MRI imaging session (TP0) was performed immediately after the bacterial inoculation. Thereafter, as soon as the MRI session was completed, 3×10^7^ granulocytes (in 0.5 ml 0.9% NaCl) were injected into the tail vein:

1. The experimental group (n = 10) received SPIO-labeled granulocytes.

2. The control group (n = 6) received non-labeled granulocytes.

The second MRI session (TP1) was performed 6 hours and the third MRI session (TP2) 12 hours after the injection of the granulocytes. The animals were sacrificed directly after the third MRI session. The validation of the results was carried out by histopathology and in vitro analyses of granulocytes after re-isolation from tissue.

### MRI

MRI imaging was performed on a 7T Pharmascan 70/16 MR scanner (Bruker Biospin, Ettlingen, Germany) actively shielded with 300 mT/m. Rats were anesthetized by 1–2% isoflurane at 1 L/min oxygen flow and placed in prone position. Images were acquired using a volume coil as both the transmitter and receiver coil (4 cm diameter).

For the calculation of T2-maps, two-dimensional multislice–multiecho (MSME) experiments were acquired (TR6200 ms and 16 TE increments of 8 ms, matrix 256×256; field of view (FOV): 3.5×3.5 cm, slice thickness 1.0 mm). An optimized 2D gradient echo sequence was used to acquire high-resolution T2*-weighted MR images (FLASH, TR2300 ms, TE 20 ms, flip angle 40deg). The FOV was 3.5×3.5 cm, slice thickness 1 mm. The inplane spatial resolution was approximately 150 µm. Susceptibility weighted images (SWI) were computed from the combined magnitude and phase image according to the standard SWI method [35].

Images were processed using Paravision 5.0 (Bruker Biospin, Ettlingen, Germany), NIH ImageJ and in-house developed algorithms for the calculation of T2-maps. An estimation of the local amount of labeled cells was performed using three-dimensional T2*-weighted MR images. To compare values between different animals and scan series for further segmentation, a basic pseudo normalization based on the highest and lowest scanner ADC-values was performed, without using a template and image transformation [36]. Susceptibility effects were studied mainly from two approaches, SWI and T2 relaxometry. The sensitivity and specificity of both techniques for SPIO-labeled cell detection were assessed by comparing the measurements obtained using different types of images. SPIO particles generate signal void regions on gradient echo images due to their strong magnetization. The signal void region has to be distinguished from that generated by possible small air bubbles. Since phase image contrast result in a linear relation with iron concentration, we considered only those images as SPIO-labeled cell detection that showed signal loss also obtained with T2 map.

Images were visually evaluated with the following criteria:

In T2* weighted images areas of decreased signal intensity of the soft tissue 6 and 12 hours after injection of granulocytes were compared with TP0-images. Well delimited collections of signal intensity (SI) losses in T2*-weighted images, with or without a relatively hypo-intense peripheral margin, were considered indicative of accumulation of SPIO.

For a semiquantificative evaluation of the SI loss the relative signal intensity over time was calculated in each group. For this purpose relative SI was determined by dividing the SI of infected area by the SI of noninfected area on T2*-weighted GRE images before and after USPIO application over time.

### Histopathology

The infected soft tissue of 6 rats in the experimental group and 4 rats in the control group were excised in toto, fixed in 4% formaldehyde for 48 hours, transversely cut in 4 µm slices and analyzed after Prussian blue staining.

Prussian blue staining is specific to detect the presence of iron in histologic specimens. Iron deposits in tissue are visualized as blue deposits. [21], [22], [26], [28].

#### Re-isolation of SPIO-labeled cells from infected tissue

The infected tissue of 4 rats in the experimental group and 2 rats in the control group were dissociated mechanically, washed with PEB (a solution containing phosphate-buffered saline (PBS), pH 7.2, 2 mM EDTA, 0.5% bovine serum albumin) and filtered. The single cell suspension was then filtered with a PreSep filter (Miltenyi Biotec GmbH, Bergisch Gladbach, Germany) prior to enrichment of SPIO-labeled cells by magnetic cell separation.

## Results

**Figure 1:**
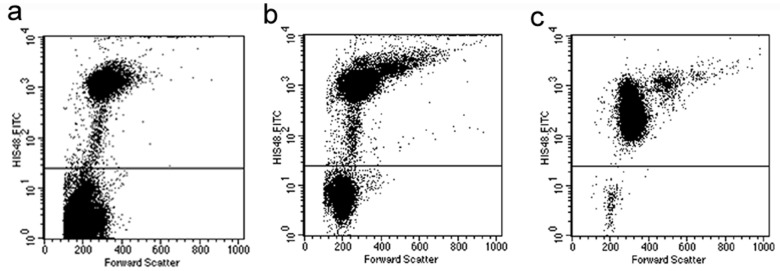
Granulocyte enrichment from rat whole blood. Granulocytes were stained using FITC-labeled HIS48 antibody and were examined by flow cytometry. (A) Granulocytes constituted 3% of the whole blood fraction before enrichment. (B) Granulocyte fraction increased to 57% after dextran sedimentation of erythrocytes. (C) Purity of granulocytes was 98% after density gradient centrifugation and removal of residual erythrocytes with the red blood cell lysis solution.

**Figure 2:**
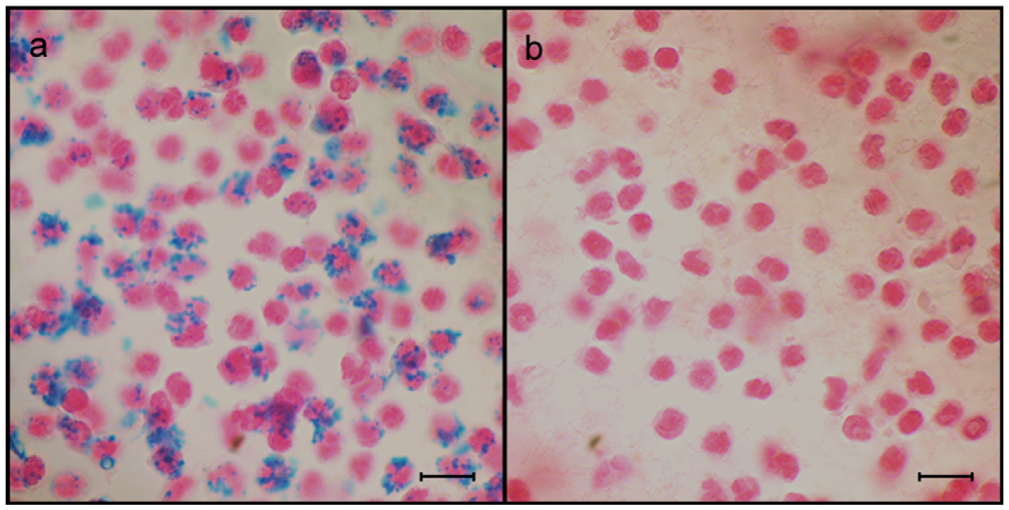
Histological verification of SPIO labeled granulocytes distribution. (A) Granulocytes were transfected *in vitro* with SPIO particles. Transfection efficiencies reached up to 90% as estimated by Prussian blue staining. Iron particle presence is identified as blue dots within cells. The scale bar equals 20 µm. (B) Unlabeled control granulocytes. Cells were counterstained with Nuclear Fast Red. The scale bar equals 20 µm.

Granulocytes were isolated from whole blood of donor rats in several steps. The final purity of granulocytes was 98%, tested by flow cytometry (Fig. 1). After transfection with SPIO-particles (FeraTrack^TM^), 90% of granulocytes were labeled as estimated by Prussian blue staining (Fig. 2). The viability of labeled and unlabeled cells did not differ significantly in cell culture. SPIO-labeled granulocytes had a viability of 88%, whereas non-labeled granulocytes had a viability of 92%.

**Figure 3:**
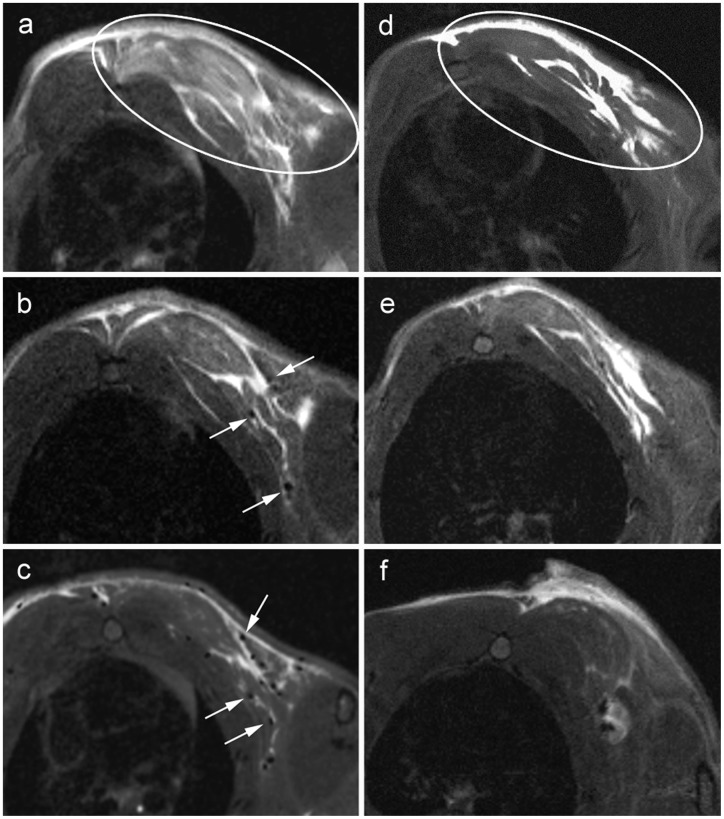
Detection of experimentally induced subcutaneous inflammatory process. T2*-weighted MRI axial images of the left pectoral area of infected rats. (A–C) Inflammatory area in the left pectoral region (ellipse) directly after inoculation of the staphylococcal suspension and (A) before injection of SPIO-labeled granulocytes, (B) 6 h after injection of the SPIO-labeled granulocytes including several spots with significant loss of signal intensity inside the inflamed area (white arrows). (C) Inflammatory area in the left pectoral region 12 hours after injection of SPIO-labeled granulocytes including multiple single spots (white arrows) with significant loss of signal intensity inside the inflamed area. (D–F) Inflammatory area in the left pectoral region (ellipse) of a control animal directly after inoculation of the staphylococcal suspension and (D) before injection of non-labeled granulocytes, (E) 6 h and (F) 12 h after injection of the non-labeled granulocytes. Areas of signal intensity loss could not be detected at any time in the control animals.

Rats were infected with *S. aureus* subcutaneously in the left thorax. *S. aureus* suspension distributed partly to right side of the thorax and to the left axillary region (Fig. 3). Thereafter the first MRI session (TP0) was performed followed by intravenous application of isogenic SPIO-labeled granulocytes into the tail vein.

**Figure 4:**
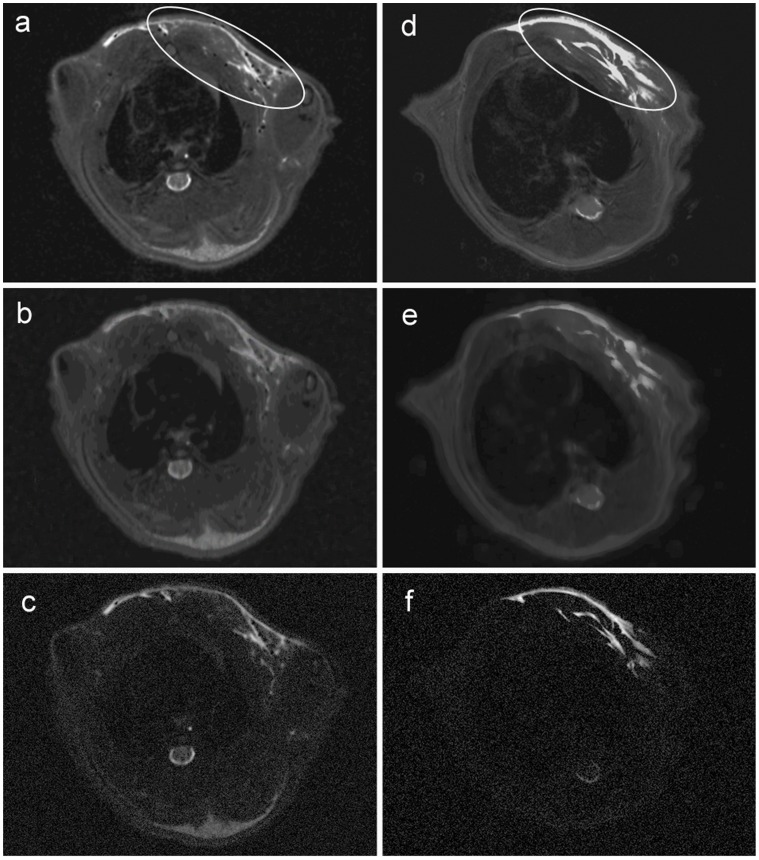
Comparison of MRI axial images of the inflammatory area. Different MRI image types 12 hours after bacterial inoculation (ellipse). (A–C) Representative images of a rat which received SPIO-labeled granulocytes. (D–F) Representative images of a rat from the control group which received unlabeled granulocytes. (A, D) T2* weighted images; (B, E) computed susceptibility weighted images (SWI); (C, F) T2-mapping computed images. Maximal loss of signal intensity was detected in T2* weighted images (D).

**Figure 5:**
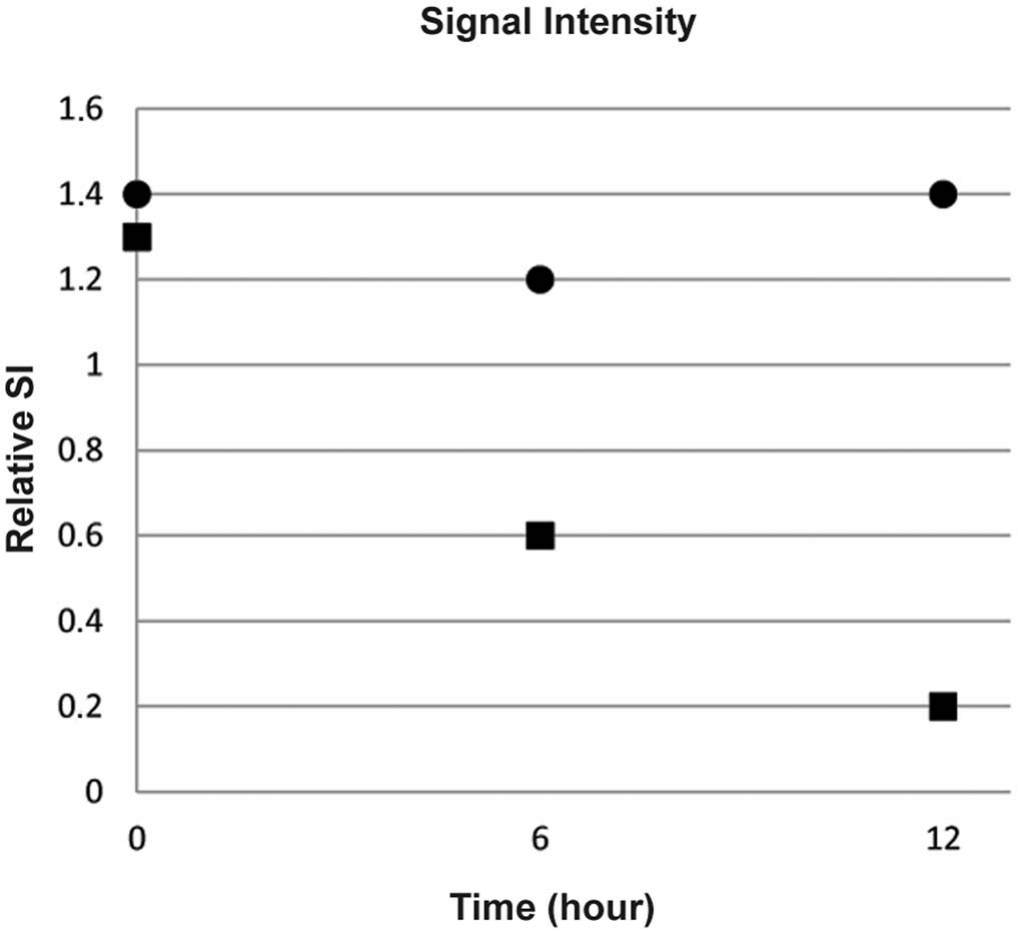
Comparision of Relative Signal Intensity over time. The relative SI was calculated by dividing SI of infected area by SI of noninfected area on T2*-weighted GRE images before (t = 0 h), after 6 and 12 h following the application of granulocytes (median values). Squares: experimental group receiving SPIO-labeled granulocytes. Circles: control group receiving unlabeled granulocytes. The difference of relativ SI over time was most prominent 12 h after SPIO application.

First SI losses in T2* weighted images were detected 6 hours after injection of the SPIO-labeled granulocytes in all animals of the experimental group. For verification of SI loss several image types (T2*, T2-map, SWI) were compared (Fig. 4). Maximal SI loss was detected 12 hours after injection of 3×10^7^ SPIO-labeled granulocytes in T2* weighted images (Fig. 3c). The semiquantifitative analysis of the relative SI loss over time demonstrated a reduction of relative SI from 1.3 to 0.2 within 12 h following the application of SPIO-labeled granulocytes, whereas the relative SI remained constant in the control group. This difference in relative SI loss between both groups was most prominent 12 h after the application of granulocytes (Fig. 5). Differences in the time course of SI loss were not observed between all animals of the experimental group. SI losses could be detected even when the number of injected SPIO-labeled granulocytes was reduced to 12×10^6^ cells per animal (data not shown). In control animals, the infection site became clearly visible as a hyperintense area in T2-weighted images, whereas SI losses could not be observed at any time point (Fig. 3d–f).

**Figure 6:**
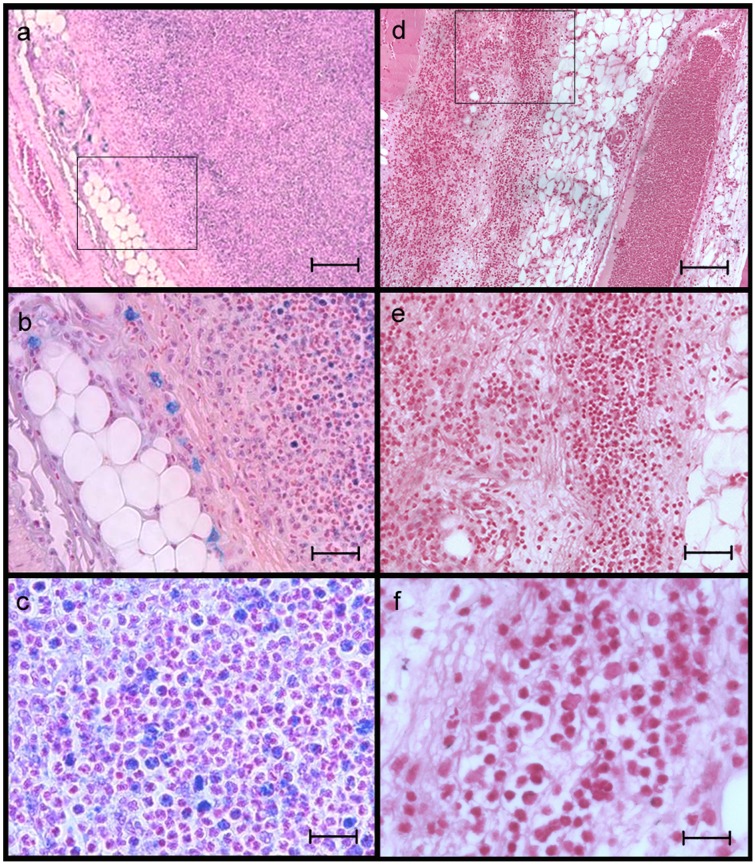
Histological sections of the infected tissue. (A–C) Prussian blue staining of infected tissue samples from rats that received SPIO-labeled granulocytes. SPIO-labeled granulocytes (blue) accumulated within the infected tissue together with unlabeled granulocytes. The scale bar equals in (A) 250 µm, (B) 100 µm, (C) 25 µm. (D–F) Prussian blue staining of infected tissue samples from control rats. Massive infiltration of unlabeled granulocytes into the whole infected region. The scale bar equals in (D) 250 µm, (E) 100 µm, (F) 25 µm.

Detection of infiltration of SPIO-labeled granulocytes in infected tissue was verified by histological analyses using Prussian blue staining (Fig. 6). The localization of SPIO-labeled cells within tissue sections correlated with the arrangement of SI losses in the MR measurements. SPIO-labeled granulocytes accumulated together with native granulocytes in the subcutaneous adipose tissue.

**Figure 7:**
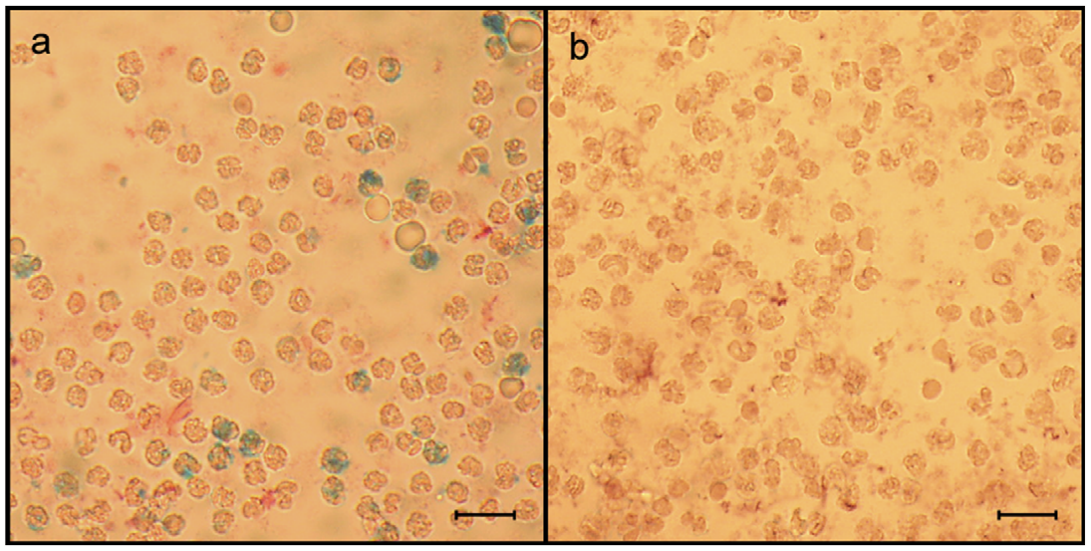
Verification of SPIO-labeled granulocytes isolated from infected tissue. (A) Fraction of granulocytes isolated from infected tissue of rats treated with SPIO-labeled granulocytes. SPIO-labeled granulocytes (blue) were identified by Prussian blue staining. (B) fraction of granulocytes isolated from infected tissue of rats from the control group, which were treated with unlabeled granulocytes. Re-isolated granulocytes are completely negative for Prussian blue staining. The scale bar equals 20 µm.

SPIO-labeled granulocytes from infected tissue were detected using flow cytometry analysis and separation by magnetic beads. The mechanical dissociation of the infected tissue recovered on average 9×10^6^ cells per animal with a fraction of >90% granulocytes. About 2×10^5^ SPIO-labeled granulocytes could be enriched via magnetic cell separation in the experimental group, resulting in a fraction of approximately 0.67% iron loaded granulocytes. In the control group, on average 2.5×10^4^ cells were eluted from the column indicating unspecific binding of 0.08% unlabeled cells. The Prussian blue staining of cells isolated by magnetic cell separation identified SPIO-labeled granulocytes only in animals from the experimental group, but not in any animal from the control group (Fig. 7).

## Discussion

Our actual study constitutes the additional step in a long way to the optimal diagnostic tool detecting infections as sensitive and specific as possible. Several previous papers have demonstrated the abiliy of cellular MR imaging to detect infectious processes [23], [37], [38]. Neutrophil granulocytes are the first phagocytizing cells that infiltrate infectious tissue [39], [40]. Here, we use these cells as molecular carriers for a magnetic field sensitive contrast agent to flag an infection process in the initial phase.

FeraTrack^TM^ SPIO-particles are dextran-coated, biodegradable and approved for in vivo human use by the US Food and Drug Administration as an MRI contrast agent [41]. They are phagocytized by cells of the reticuloendothelial system and their iron is rapidly incorporated into hemoglobin of erythrocytes [41], [42].

The iron oxide labeling method used in this study enables a fast and effective load of neutrophils resulting in a high final transfection rate. This is important, because of the short half life of neutrophils, especially after *in vitro* cell separation. In contrast to humans, the proportion of granulocytes from whole circulation leukocytes averages to only 20% in rats. Therefore, G-CSF was used additionally to maximize the neutrophil yield per animal [30].

In vitro labeling is labor-intensive and implies the hazard of cross infection during the handling of autologous blood in the course of in vitro cell labeling [43]. An additional drawback is that the imaging signal is not directly linked with the cell viability and persists after cell death. Iron oxide released from cells due to other mechanisms could also result in a similar signal [41]. Furthermore, effects of labeling on cell function and viability need to be considered. Iron labeling of various cell-types have not been shown to have any toxic effects with regard to cell proliferation, viability or differentiation potential [42], [46], [47]. The specific accumulation of the injected SPIO-labeled granulocytes into the infected lesion in our study proves their preserved chemotactic properties and functionality for reaching their final destination. Daldrup-Link et al. injected ferumoxide-labeled human hematopoietic progenitor cells into athymic mice intravenously in order to evaluate their *in vivo* distribution. The iron-labeled hematopoietic cells caused signal intensity loss in liver, spleen and bone marrow. Additionally, the SI decrease was significantly stronger after inejection of iron oxide-labeled cells compared to controls that received injections of the pure contrast agent [48].

SI losses in T2*-weighted MR images were only determined in animals of the experimental group, which received SPIO-labeled granulocytes. The quantification of relative SI loss showed that the difference between groups was most prominent after 12 hours following SPIO application. SPIO-particles cause MR signal reduction due to its susceptibility effect reducing T2 and T2* values in the tissue [49]. However, SPIO concentration in the tissue, magnetic field strength, and intrinsic characteristics of the SPIO particle affect the signal reduction [50], [51].

SPIO particles' relaxation rates differ whether they are localized within cells or within regions of freely diffusible water. T2* weighted gradient echo acquisitions provide the best possible sensitivity to detect intracellular SPIO particles [46]. The susceptibility effect on the SPIO-particle extends well outside the volume occupied by the cell. This extension augments its detectability. While the iron amount is mostly sufficient for MR detection a heterogeneous or hypointense background is problematic. Gradient echo T2* weighted measures are sensitive to background field inhomogeneities induced by blood, imperfect shimming, and ferritin deposits. Thus, they have poorer specificity for iron particles. T2 weighted spin echo acquisitions can be much less sensitive to iron labeled cells than T2* sequences [46]. T2 and T2* based imaging methods depict SPIO labeled cells as areas with signal loss. Deoxyhemoglobin in small vessels also generates hypointensities similar to the SPIO labeled cells, making it potentially difficult to differentiate between transplanted cells and slow flowing blood. Administration of paramagnetic contrast agent prior to imaging could improve the detection of labeled cells [47]. Nevertheless, differentiation between the signal loss caused by the intracellular nanoparticles and native low signals, for example those from artifacts or imaging metals such as calcium is challenging. The MRI sequences were chosen as a compromise between imaging time and sensitivity, sacrificing the use of intensive quantitation of imaging values. Nevertheless, semiquantitative analysis of image data is possible as shown. Furthermore, the detection of labeled cells is limited by partial volume effects, in which void detection is dependent on the resolution of the image.

Compared to other imaging modalities such as positron emission tomography (PET), single-photon emission computed tomography (SPECT) or optical fluorescence imaging, MRI has traditionally been thought of as having a low sensitivity. The majority of studies utilizing MRI for the detection of SPIO labeled cells have only examined large groups of cells (10^6^–10^7^) [52]–[54].

For the analysis of the migration patterns of cells the ability to detect smaller groups of cells would provide a more accurate picture of the fate of transplanted cells. First i*n vitro* detection of single cells using MRI was demonstrated by Dodd et al. for SPIO-labeled T cells in gelatin [55]. By scanning at high resolution (25 µm isotropic), using optimized RF coils, long scan times (2 hours) and high field (7 Tesla) to achieve adequate signal to noise ratio (SNR) the authors met the requirements for cell detection. To perform cell detection *in vivo*, images with high resolution and high SNR have to be achieved under less optimal imaging conditions. Hoehn et al. reliably demonstrated an in vivo detection limit of 500 embyronic stem cells implanted into the brain of rats and imaged at 7 Tesla [56]. Kircher et al. showed that as few as three SPIO labeled cytotoxic lymphocytes/voxel could be detected at 8.5 T in tumors in live mice [57]. In our setting, we reduced the number of injected granulocytes to 12×10^6^ cells per animal and still obtained significant SI losses. However, it is not possible to deduce the actual number of labeled granulocytes causing each spot of SI loss in MRI in the infected area. Therefore, a follow-up study is needed to determine the minimal quantity of cells required for a certain diagnosis.

### Limitations

Two main limitations can be recognized in the MR methodology. Normalization of the MRI signal with respect to changes in image noise was performed by simply subtracting the background intensity from the tissue signal. However, since MRI noise is Rician distributed and not additive, this simple technique may not be able to correctly estimate the true MRI signal. More complex signal estimations may be more appropriate to improve this aspect.

A second limitation is the possible presence of field inhomogeneities and distortions that can affect the T2* value measurements in different regions. Automatic and local shimming of the system should have reduced these effects. However, shim correction cannot fully compensate for sharply varying inhomogeneities. In summary, we have developed a unique form of cellular MRI technology also suited to clinical MR field strengths, and have demonstrated high detection without radiation and toxic tracers necessary in other imaging modalities.

Diverse limitations of our feasibility study have to be addressed. The experiment was performed in a small number of animals restricting our statistical analysis. The specificity of MRI is markedly diminished in the postoperative setting. Therefore, further research has to focus on chronic infections associated with more macrophage and lymphocyte recruitment, because of the rather low prevalence of granulocytic processes in the chronic setting [22], [28]. A pan-leukocytic labeling with SPIO-particles might be useful in chronic settings. It should be taken into consideration that SPIO-loaded cells may die on their target location as granulocytes have a quite short half-life. These cells can be detected by MRI and Prussian blue staining even after sacrifice of their biological function. Even macrophages incorporating dead cell debris stain positive for Prussian blue as they contain the SPIO-particles and cause SI loss in MRI [58].

It is crucial to figure out the specificity and sensitivity of the herein presented method and compare it with other intravenous MRI contrast agents [59], [60].

### Conclusion

We conclude that intravenously applied SPIO-labeled granulocytes migrate to the infection site and cause MRI signal loss in acute infections. Our findings are the basis for further investigations in order to push forward the non-invasive detection of infectious processes. It remains to be demonstrated, whether these findings can be transferred to subacute and chronic infections and/or differentiate possible infection from sterile physiological postsurgical healing processes in humans.
